# Biomimetic Design of Soil-Engaging Components: A Review

**DOI:** 10.3390/biomimetics9060358

**Published:** 2024-06-14

**Authors:** Zihe Xu, Hongyan Qi, Peng Gao, Shuo Wang, Xuanting Liu, Yunhai Ma

**Affiliations:** 1 The College of Biological and Agricultural Engineering, Jilin University, 5988 Renmin Street, Changchun 130025, China; zhxu21@mails.jlu.edu.cn (Z.X.); qhy18@mails.jlu.edu.cn (H.Q.); penggao15@mails.jlu.edu.cn (P.G.); wangshuol78@163.com (S.W.); xuantingl20@mails.jlu.edu.cn (X.L.); 2 The Key Laboratory of Bionic Engineering, Ministry of Education, Jilin University, 5988 Renmin Street, Changchun 130025, China

**Keywords:** biomimetics, soil-engaging components, draught reduction, anti-adhesion, wear resistance

## Abstract

Soil-engaging components play a critical role in agricultural production and engineering construction. However, the soil-engaging components directly interacting with the soil often suffer from the problems of high resistance, adhesion, and wear, which significantly reduce the efficiency and quality of soil operations. A large number of featured studies on the design of soil-engaging components have been carried out while applying the principles of bionics extensively, and significant research results have been achieved. This review conducts a comprehensive literature survey on the application of biomimetics in the design of soil-engaging components. The focus is on performance optimization in regard to the following three aspects: draught reduction, anti-adhesion, and wear resistance. The mechanisms of various biomimetic soil-engaging components are systematically explained. Based on the literature analysis and biomimetic research, future trends in the development of biomimetic soil-engaging components are discussed from both the mechanism and application perspectives. This research is expected to provide new insights and inspiration for addressing related scientific and engineering challenges.

## 1. Introduction

Soil is an important resource that supports biodiversity, environmental stability, and agricultural production [1]. With the development of society and the improvement of the levels of industrialization, various kinds of machinery have been designed and manufactured to carry out soil-related operations. These machines have found widespread applications in agriculture, civil engineering, and transportation, significantly enhancing operational efficiency. The working efficiency and quality of these machines often depend on their soil-engaging components. Through the mechanical interaction between these components and the soil, the effect of contacting, cutting, crushing, turning, or transporting the soil is produced [2]. However, soil comprises solid particles, liquid, and gas, which constitute a three-phase system with complex physical and mechanical properties. This means that soil-engaging components, in addition to overcoming resistance during operation, have to face the negative effects caused by soil adhesion and wear. For example, in agriculture, tillage components require 30–50% of energy consumption to overcome soil friction and adhesion [3]. Wear between the soil and soil-engaging components also reduces the operating efficiency and service life of the components, further increasing production costs [4]. Therefore, it is crucial to improve or optimize the design of soil-engaging components to solve these problems.

Throughout the long process of evolution, numerous animals and plants in nature have ingeniously shaped their structures and functions to adapt to the constantly changing environment [5]. These organisms serve as rich sources of inspiration for researchers. In recent years, with the continuous development of bionics, bionic design methods have been widely used in soil-engaging component research [6]. By observing and analyzing the structures/behaviors of organisms, and through different design methods, scholars have developed soil-engaging components with excellent biological properties [7]. For instance, soil-dwelling animals, such as mole crickets, moles, and badgers, exhibit strong digging capabilities and high soil-loosening efficiency. Inspired by this, researchers have designed excavation components with low resistance characteristics [8,9,10]. Similarly, the surface structures of organisms like shells, pangolins, and dung beetles have inspired the development of components aimed at reducing adhesion and increasing wear resistance [11,12,13]. These biomimetic designs effectively enhance the performance of components and improve the quality of operations.

However, previous reviews in this field have typically focused on a limited scope, such as specific components or applications. In addition, innovative and impactful studies continue to emerge as more researchers become involved. A comprehensive review is needed to provide a thorough understanding of the current state of the research field. Various types of soil-engaging components operate in distinct environments and modes, resulting in diverse selections of bionic prototypes and applications of bionic elements. Through optimizations in biomimetic design, these components manage to enhance performance in one or several aspects. Therefore, this paper presents an informative literature survey from the perspective of biomimetic soil-engaging components achieving superior performances in different aspects. Firstly, this article mainly reviews the research content of three types of biomimetic soil-engaging components with low-resistance, anti-adhesion, and wear resistance properties. Subsequently, it summarizes the methods and mechanisms of biomimetic soil-engaging components. Finally, based on the literature analysis, further applications and trends in biomimetic design for soil-engaging components are discussed. The research is expected to inspire future researchers to develop novel techniques for enhancing the performance of these components.

## 2. Biomimetic Soil-Engaging Components

Soil-engaging components vary significantly in form and structure across different engineering fields. In agricultural machinery, there are many types of soil-engaging components. These include plows, subsoilers, rotary blades, and press rollers used to prepare the soil before sowing; stubble breakers, furrow openers, and soil coverers employed in planting operations; and components used to dig underground crops during harvesting operations [14,15]. In civil engineering, the main soil-engaging components include excavation buckets, bulldozing plates, and drills in earth-moving machines [16,17]. The soil-engaging components related to vehicles are mainly wheels and tracks [18]. The above-mentioned components usually encounter some common problems during operation, such as high operating resistance, serious soil adhesion, and severe wear. Biomimetic designs offer innovative solutions to optimize performance. Based on the optimized performance of the components and the available literature, they are classified into the three categories of low-resistance, anti-adhesion, and wear resistance.

### 2.1. Low-Resistance Biomimetic Components

Soil-engaging components consume a large amount of energy to overcome the resistance created during tillage, digging, and drilling operations. Thus, reducing resistance has become an important goal in the optimization of soil-engaging components [19]. The structures or behaviors of organisms inspire the design of low-resistance soil-engaging components, which subsequently provide the basis for component improvement and optimization. Low-resistance soil-engaging components inspired by biological structures and motions will be discussed in the following sections.

#### 2.1.1. Components Inspired by Biological Structures

**Figure 1:**
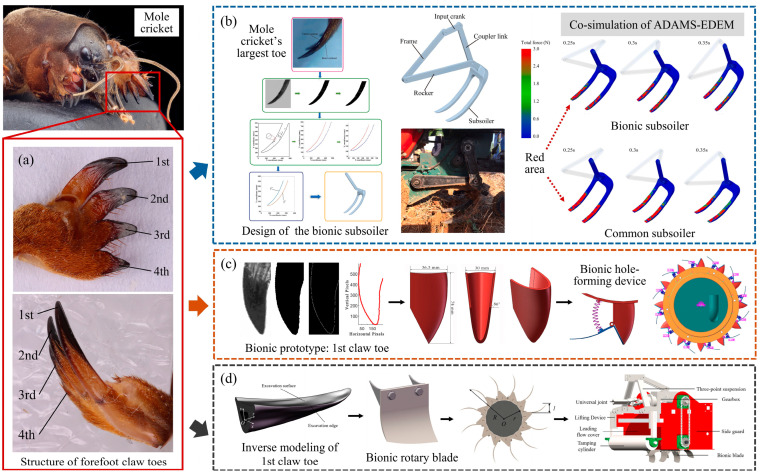
Low-resistance soil-engaging components inspired by mole crickets. (**a**) Structure of the claw toes on the forefoot of the mole cricket [24], (**b**) tea garden bionic subsoiler [8], (**c**) bionic rotary blade [24], (**d**) bionic fixed hole-forming device [20].

Organisms have evolved over millions of years and have shown good adaptability to complex survival environments. Animals such as mole crickets, moles, and badgers have remarkable digging abilities in their legs, feet, claws, and teeth. Therefore, these structures were selected as bionic prototypes for the design of low-resistance soil-engaging components. Their digging organs have special curves and wedge-shaped structures which can effectively reduce digging resistance and improve digging efficiency. Fossorial animals were the first considered for bionic draught reduction designs. Mole crickets are typical soil-dwelling creatures. They live in burrows in the soil for a long time. The front legs have been modified into digging feet with a unique structure (Figure 1a), which shows strong soil-digging capabilities. Researchers have successfully extracted the contour of mole crickets’ claw toes, applying it to the design of soil-engaging components to achieve significant draught reduction effects.

Zhang et al. [8] processed the image of the largest toe of a mole cricket’s forefoot, scaled and rotated the inner and outer contour curves, and designed a bionic subsoiler for loosening the soil in the tea garden. The design is illustrated in Figure 1b. During the subsoiler operation, the horizontal and vertical resistance decreased by 16.34% and 24.53%, respectively, leading to a 9.64% reduction in entire machine energy consumption. The red area (Figure 1b) shows the cloud diagrams of the force on the surface of the bionic subsoiler and common subsoiler. It visually demonstrates that the bionic subsoiler is subjected to less force at the same moment. Wang et al. [20] also designed a bionic hole-forming device for seeding by image processing with contour curve fitting, as shown in Figure 1c. Through simulation analysis, it was found that the velocity distribution of soil particles on the bionic curve was more uniform, which made the bionic hole-forming device show a smaller soil penetration resistance than both traditional square- and cone-shaped hole-forming devices, measuring only 7.51N. At the same time, the disturbance of the soil caused by the device was also reduced as the resistance was reduced. In addition, the bio-inspired soil-engaging components developed by applying the contour features of mole crickets’ claws to the excavator bucket teeth, furrow openers, and rotary blades also achieved good drag reduction performance [21,22,23].

It is worth noting that the extraction of contour curves from two-dimensional images can be influenced by factors like shooting angle and image quality, potentially leading to inaccuracies. Three-dimensional reconstruction can often extract contour curves more accurately. Zhu et al. [24] reconstructed the three-dimensional model of the claws of the mole cricket’s front feet. Based on this, they extracted the contour curves of the edge and the excavation surface. Then, they designed a bionic rotating blade for digging Cyperus esculentus, as shown in Figure 1d. Through adjustments to blade arrangement and operational parameters, the optimal excavation performance of the bionic blade was investigated. The advantages demonstrated by these components are attributed to the use of the unique contour curve of the claw toe. The characteristics of the curvature change in the bionic curve make the soil being cut move quickly along the digging surface, avoiding the accumulation of soil. In addition, the pressure on the surface of the component can also be improved, thereby achieving low cutting and digging resistance in the soil.

**Figure 2:**
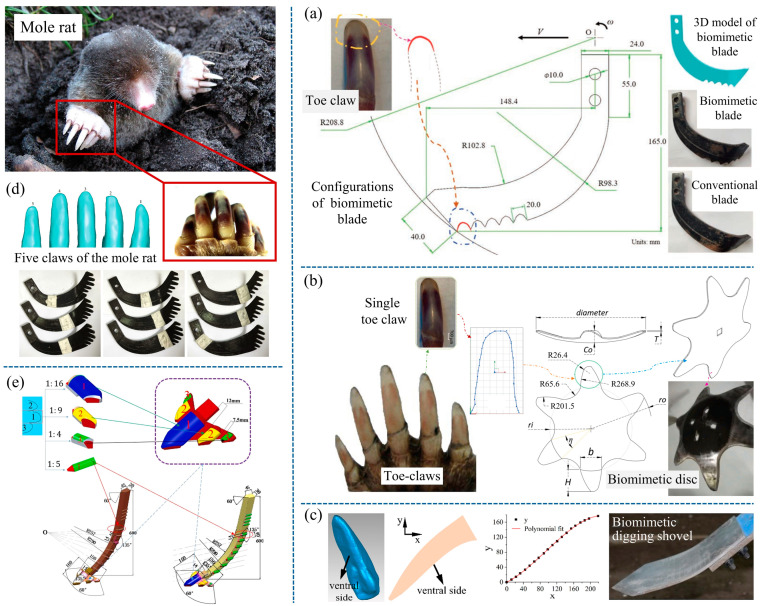
Low-resistance soil-engaging components inspired by mole rats. (**a**) Biomimetic tiller blade [29], (**b**) biomimetic disc [30], (**c**) biomimetic potato digging shovel [32], (**d**) biomimetic rotary tillage blades based on five fore claws of mole rat [34], (**e**) bionic inspired subsoiler [35].

Mole rats are famous for their outstanding soil-digging ability. They use the five claws of their forefoot as their main digging tool, working in tandem with their hind limbs to move quickly through the soil while digging [25,26]. Numerous researchers have drawn inspiration from mole rats’ claw toes for the design of soil-engaging components. Ji and Tong et al. [9,27,28] studied the drag-reducing characteristics of the claw toes of mole rats and found that the inner surface of the claw toes can guide the soil to move on the surface and produce rapid crushing. Based on this, a bionic rotary blade was designed. Through testing, it was found that the average torque of the bionic rotary blade was reduced by 3.14 Nm. Similarly, Torotwa et al. [29] also designed a bionic rotary tiller blade based on the claw toe of a mole rat, as shown in Figure 2a. Results revealed that the biomimetic blade torque was reduced by 21.05%, the soil was cut into smaller clods, and the straw burial rate and the quality of the seedbed after operation were improved. Li et al. [26] extracted the contour curve of the tip of the second claw toe of a mole rat and designed a biomimetic cutting disc. The results showed that the biomimetic disc cut the soil with less resistance than the conventional disc cutter, and the crushing effect on the soil was enhanced. Torotwa et al. [30] also designed a biomimetic disc based on the mole’s second claw toe, as shown in Figure 2b. This component was tested in an indoor soil bin covered with straw. The results showed that the mean vertical resistance of the biomimetic disc was reduced by 21.4%, the mean draught force was reduced by 28.7%, and the efficiency of the cutting straw was improved at an operating speed of 1 km/h. In the meantime, they designed a curved-toothed disc inspired by the arc-shaped structure of the mole’s claw. The curved-toothed disc not only met the drag reduction performance but also further improved the straw-cutting efficacy by 26.31% [31].

The above are all biomimetic components used for cutting soil. The cutting edge with the contour curve of the mole claw toe increases the penetration performance of the tool. This allows the component to penetrate and cut the soil with minimal resistance, thus reducing the resistance or torque of the component. Yu et al. [32] developed a digging shovel for potatoes. As shown in Figure 2c, the digging shovel has a bionic macro-surface structure. The test results show that the biomimetic potato digging shovel exhibited significant resistance reduction characteristics, with a maximum reduction of more than 8.41%. Furthermore, the draught force was reduced by 13.33% when multiple shovels were used in combination. The draught reduction in the biomimetic digging shovel was because of the bionic curved surface. This surface caused the soil to move upward, constantly changing the motion state and stress state of the soil, thereby facilitating easier soil fragmentation.

Some researchers have found that the multi-claw structure of mole rats also plays a key role in efficient digging. The five claws of a mole’s hand are in the same plane during excavation, working collectively to enhance soil-cutting performance and diminish the formation of soil wedges in the forward direction [25,33]. On this basis, Yang et al. [34] designed a rotary tillage blade with a multi-claw combination structure, as shown in Figure 2d. Field tests showed that bionic blades equipped with multi-claw structures exhibited lower horizontal resistance and torque compared to conventional blades. This reduction is attributed to the rotary blade’s collective action, which allows for soil slip cutting at a smaller inclination angle, thereby minimizing frictional resistance and retaining the low-resistance soil penetration characteristics of a single claw toe. Song et al. [35] built a bionic subsoiler by extracting the middle three-toe structure of the mole’s claw toe and applying it to the tine of a standard subsoiler, as shown in Figure 2e. The subsoiler has a significant drag reduction effect, while the width of the disturbance to the plough pan is increased by up to 20.6%. In addition to moles, the claw toes of voles [36,37], house mice [38], and muskrats [39], which are small animals that are good at digging, have also been found to have low resistance characteristics. Scholars have applied their bionic curves in subsoiler shanks to deal with the problem of high draught forces in subsoiling operations. This allows subsoiling equipment to be towed by smaller-horsepower machinery, alleviate soil compaction, and improve soil productivity to a certain extent.

**Figure 3:**
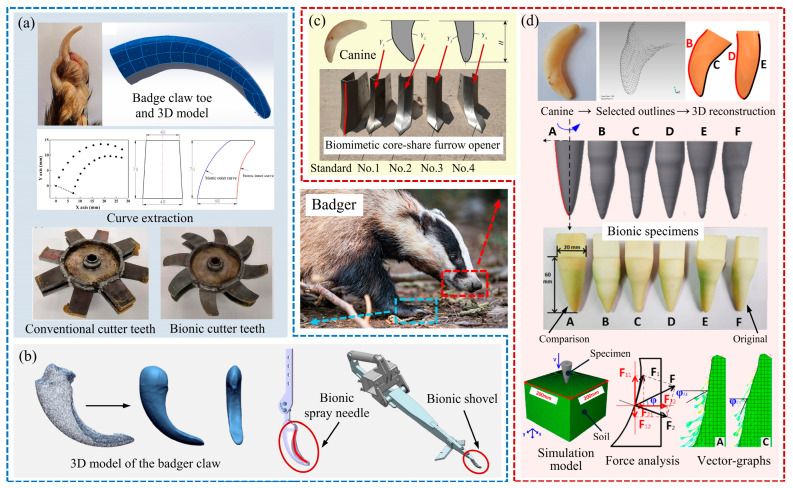
Low-resistance soil-engaging components inspired by badge claw toe/canine. (**a**) Bionic cutter teeth [10], (**b**) bionic shovel and bionic liquid fertilizer deep application spray needle [43,44], (**c**) biomimetic core-share furrow opener [48], (**d**) bionic curves specimens for test soil penetration resistance [47].

The badger also has great digging ability, with stout limbs and long, curved clawed toes used to cut compact soil for burrowing or foraging [40]. Compared with other organisms in the soil, badgers are very suitable as bionic prototypes for soil-engaging components due to their larger body size, which makes it easier to observe and measure features. Guan et al. [10] established a three-dimensional surface model of the badger’s claw toes via 3D scanning. They extracted the internal and external contour curves of the claw toes and designed bionic cutter teeth with an asymmetric structure. This structure is narrow at the top and wide at the bottom, as shown in Figure 3a. Finite element simulation concluded that bionic cutter teeth are significantly better than conventional cutter teeth in reducing cutting resistance and improving soil fragmentation. This superiority is attributed to the bionic curve profile of the cutter, which exhibits better sliding cutting performance, thereby reducing frictional resistance during penetration into the soil and cutting energy consumption. Ma et al. [41] designed biomimetic excavator bucket teeth using reverse engineering and 3D-printing techniques. They utilized the longitudinal profile curve of a badger claw toe as a bionic prototype. The penetration test was conducted on the sample of reduced bucket teeth made of acrylonitrile butadiene styrene (ABS) material. The resistance of the sample decreased by up to 12.6% compared to the standard bucket teeth of type 80, observed when the depth of penetration ranged from 10 mm to 40 mm. Furthermore, Akter et al. [42] took both longitudinal and vertical cross-sectional curves of the claw toe into consideration and designed bionic excavator bucket teeth that contained more characteristics of the dog and badger claw toes, which also had good drag reduction effects.

The above-mentioned badger-inspired soil-engaging components are constructed based on curves. Zhou et al. [43,44] obtained a three-dimensional model of a badger claw. After magnifying it by 10 times, it was directly used in the design of a liquid fertilizer deep application spray needle and a carrot harvesting ripper, as shown in Figure 3b. The results showed that, under appropriate operating parameters, these components could considerably reduce resistance and energy consumption. The discrete element method (DEM) is a commonly used and effective method for analyzing soil−component interactions [45]. It was also found that, by DEM, that soil particles can move with variable acceleration along the bionic curved surface. This allows components with three-dimensional bionic structures to reduce energy loss when breaking soil. Such a direct bionic design method ensures that the components contain the most structural characteristics of the claw toe. Low-resistance and low-friction characteristics of claw toes can be effectively replicated on soil-engaging components. However, there are differences between various claw toes, and between distinct individuals, which is an issue that needs to be considered during design.

It is noteworthy that badger teeth are extremely hard, sharp, and also have excellent cutting capabilities [46]. Ma et al. [47] studied the process of badger teeth penetrating into the soil, as shown in Figure 3d. The simulation results demonstrated that ABS-material bionic specimens designed with the badger canine outlines could change the force direction, thereby reducing the penetration resistance. Consequently, it is worth trying to use badger teeth as bionic prototypes to design low-resistance soil-engaging components. Jia et al. [48] applied the contour curves on different positions of badger teeth to the design of the soil-engaging components and developed four biomimetic furrow openers, as shown in Figure 3c. A soil bin test showed that they all reduced operating resistance. Among them, the opener (No. 3), designed with the side edge curve of the badger canine, achieved the best drag reduction effect at a speed of 7.2 km/h, reducing resistance by 8.71% compared to the standard opener.

**Figure 4:**
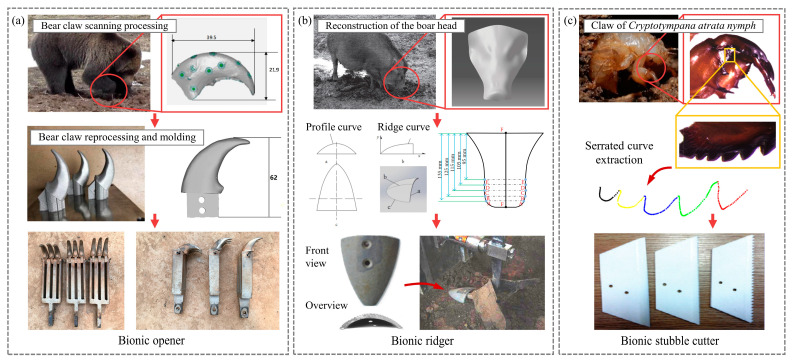
Low-resistance components inspired by soil animals. (**a**) Bionic opener inspired by bear claw [49], (**b**) bionic ridger inspired by a boar’s head [50], (**c**) bionic stubble cutter inspired by *Cryptotympana atrata nymph* [51].

The body structures of other soil animals in nature have also been mimicked for use in soil-engaging component design. For example, a soil-plowing device used bear claws as a bionic prototype [49]; a ridger imitated a boar’s head [50]; a biomimetic stubble cutter imitated the fore claw of the cicada [51]; and a biomimetic soil loosening shovel utilized an anteater’s claws [52], as shown in Figure 4. These biomimetic soil-engaging components have achieved satisfactory results in terms of drag reduction performance. However, these studies are mostly oriented to experimental results, and there is limited research on the mechanism of draught reduction in these biological contours and surfaces, which still has great research prospects.

Fish usually have streamlined bodies, which allows them to reduce drag force more effectively in the water. This is a strategy that has evolved over time in high-speed swimming organisms. Some fish have unique skin structures that further reduce the drag, such as sharks [53]. Although water and soil are two completely different environments, imitating their contours or surface microstructures has proven to be an effective method for studying soil-engaging components.

Sharks with rough skin can swim quickly in the water. Inspired by the ridging (triangle- and arc-shaped) shapes of shark skin, Zhang et al. [54] designed a bionic ridging shovel that exhibited an average drag reduction rate of 3.65%. Niu et al. [55] also designed and fabricated two bionic subsoiler tines inspired by the microstructure of shark skin. Test results showed that the subsoiler tine with discontinuous bionic microstructures experienced reductions of 21.3% and 24.8% in horizontal and vertical forces, respectively, and the bionic surface had the advantage of reducing drag and stress fluctuation. Similarly, Sun and Wang et al. [56,57] developed six types of biomimetic subsoilers. They simplified the shark skin structure into a triangular prism and cylindrical structure, which were used for the tine and shank of the subsoiler, respectively. Discrete element simulation results showed the optimal bionic element parameter of h/s (height-to-lateral-rib-spacing ratios) of 0.57. A higher value of h/s led to a better drag reduction effect. However, a h/s that was too high could also have a negative effect. Additionally, sailfish [58], tuna [59], and sturgeon [60] possess inherent speed advantages, with openers modeled after their body contours achieving substantial resistance reduction. These studies demonstrate the great potential of structural bionics in the design of soil-engaging components. However, the intricacy of these designs necessitates more complex manufacturing processes, presenting challenges in terms of cost and durability in practical applications.

**Figure 5:**
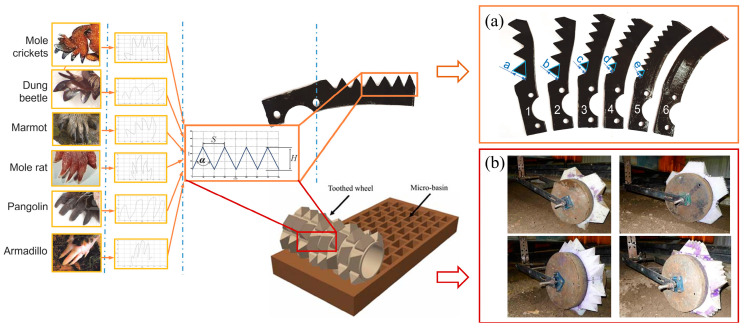
Convergent evolution inspired soil-engaging components. (**a**) Bioinspired mini rotary tiller’s blade [61], (**b**) bionic toothed wheel [62].

In conclusion, the design of low-resistance components inspired by the shape of the claws and toes, heads, teeth, body surfaces, and other parts of living creatures is currently an effective and common method. In essence, there are actually some similar characteristics to these parts. The contour curvature of the claws and toes of crickets, such as mole crickets, moles, and bears, usually changes, which can make the surface soil more susceptible to stress fluctuations and accelerated breakage. The contour tip curvature of the claw toes is also larger, making it easier for the claw toes to penetrate the soil. Zhang et al. [61,62] investigated the structures of claw toes in various soil burrowing animals from the perspective of convergent evolution, finding similar geometrical analogous serration features. Based on this, they designed low-resistance bioinspired rotavator blades and toothed wheels, as shown in Figure 5. The serrated structure had the greatest stress concentration capacity, which increased the tendency of soil failure and reduced penetrating resistance. Nevertheless, it is important to acknowledge that the current research methods based on biological structures for biomimetic design are relatively limited, mainly concentrating on linear or curved perspectives. This approach might overlook a lot of potentially influential biological feature information, leading to certain limitations in the research results. Therefore, it is necessary to systematically explore and adopt more testing or experimental methods to improve the effectiveness of future biomimetic designs of soil-engaging components.

#### 2.1.2. Components Inspired by Creature Movements

Numerous creatures exhibit effective and efficient movement through soil. In addition to the excellent capabilities of their body organ structures, their motion behaviors, such as digging, peristalsis, and rotation, that they display also play an important role. These behaviors have inspired scholars to conduct bionic design research on soil-engaging components from the perspective of dynamic bionics.

Animals specializing in soil excavation achieve efficient digging through the coordinated actions of their claws, teeth, and other specialized organs. For example, moles allow the forearms to move through their limits of motion and dig using the mechanism of humeral rotation [63]. African mole rats employ their teeth to shovel and break up soil, followed by using their limbs to push it backward, resulting in efficient excavation [64].

**Figure 6:**
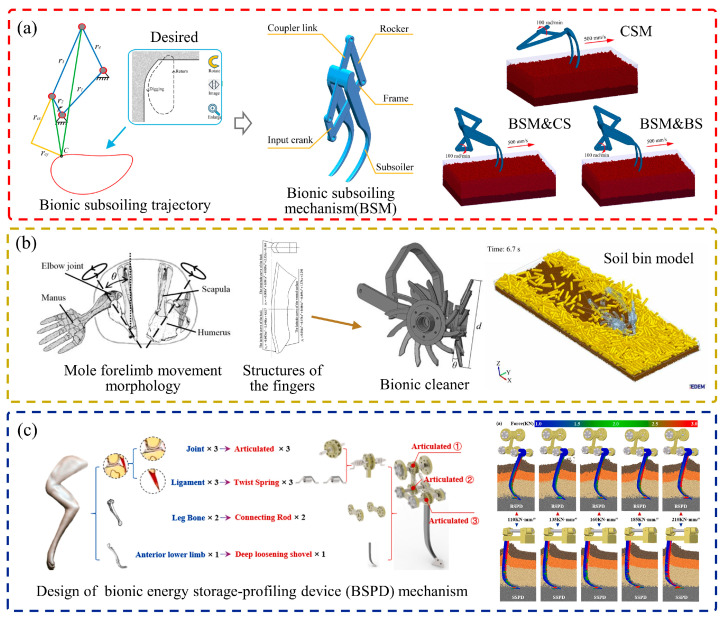
Low-resistance soil-engaging components inspired by biological excavation movements. (**a**) A four-bar subsoiling mechanism inspired by digging trajectory of the mole forefoot [65], (**b**) bionic mole forelimb row cleaners [66], (**c**) bionic energy storage-profiling device based on the fore-upper limbs of the hare [71].

Zhang et al. [65] designed a bionic subsoiling mechanism which used a four-bar mechanism to simulate the digging trajectory of a mole’s front paw with a subsoiler, as shown in Figure 6a. Comparative analysis with a conventional subsoiling mechanism revealed a 10.87% reduction in drag for the bionic subsoiling mechanism equipped with a standard subsoiler, and there was a further reduction of 18.4% when it was equipped with a bionic subsoiler. At the same time, the bionic movement of the subsoiler significantly increased soil disturbance. Jia et al. [66] also developed a bionic mole forelimb intelligent row cleaner based on the motion morphology of a mole’s front limb, as shown in Figure 6b. During operation, the fingers of the cleaners formed a biomimetic angle of θ° with the ground, cutting into the residue layer and the soil layer. Simulation analysis demonstrated that the bionic cleaner reduced drag by up to 49.4% compared to standard cleaners.

When mole crickets burrow underground, their front legs move in a unique pattern of forward digging and horizontal expansion [67]. Based on this behavior, Li [68] utilized the motion trajectory of a mole cricket’s front feet in designing a potato digging shovel, resulting in a 27.5% reduction in digging resistance. A hare’s excavation ability is equally impressive, allowing it to quickly dig through numerous, lengthy, and complex underground caverns [69]. Lu [70] designed a bionic ditch opener by combining the structure and motion of hare claw toes. By excavating the soil instead of compressing it, like traditional openers, this design minimizes resistance. Tests showed that, at a forward speed of 12 km/h and a depth of 70 mm, the resistance was reduced by 17.45%. Similarly, Zhao et al. [71] designed a bionic profiling-energy storage device based on the structure of a hare’s fore-upper limb combined with kinematic bionics, as illustrated in Figure 6c. Simulations and testing at 400 mm tillage depth showed that, compared with traditional self-excited energy storage-profiling devices, the bionic energy storage-profiling device had a significant advantage. Under different elastic stiffness values, the bionic device reduced the subsoiling resistance and the operation fuel consumption by 16.1%. In addition, it also had a higher freedom of motion and the ability to correct the change in tillage depth. The drag reduction effect of these soil-engaging components that mimic the digging behavior of animals is effective, but it also complicates the design and analysis of the components. Therefore, simulation analysis is commonly used to study the interactions between moving components and soil, but it may be affected by model accuracy and parameter calibration, leading to errors in the results.

**Figure 7:**
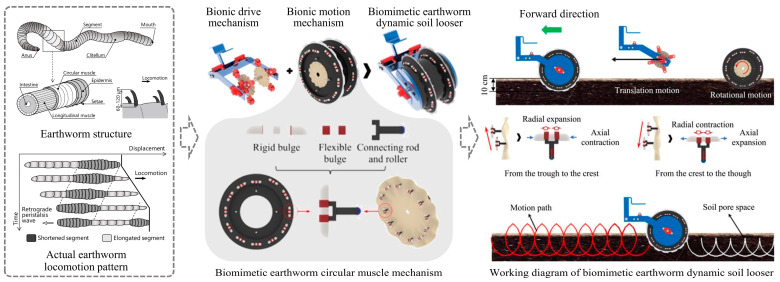
Biomimetic earthworm dynamic soil looser [75,77].

Invertebrates living in the soil usually move forward in a wriggling manner, such as earthworms and polychaete annelid worms. The wriggling motion is a special form of muscle movement that induces motion by expanding and contracting parts of the body [72]. During the process of earthworm wriggling, the segments advance in waves from the front to the back, which improves the efficiency of the earthworm’s movement in the soil [73]. Jia and Zhao et al. [74,75] designed a biomimetic earthworm dynamic soil looser consisting of a bionic drive mechanism and a bionic motion mechanism, as illustrated in Figure 7. The soil looser had biomimetic earthworm circular muscle mechanisms on its surface and was arranged in the manner of an Archimedean spiral. The experimental results showed lower resistance. When the biomimetic soil looser moved forward, it simulated the motion state of earthworm circular muscles, expanding periodically. In scenarios requiring deeper penetration depths, Naziri et al. [76] developed an earthworm-inspired subsurface penetration probe and experimented with it using a lunar regolith simulant. They found that, under the maximum peak power demand of 0.2 watts at a constant speed of 0.2 cm/s, the penetration resistance was reduced by 80%.

**Figure 8:**
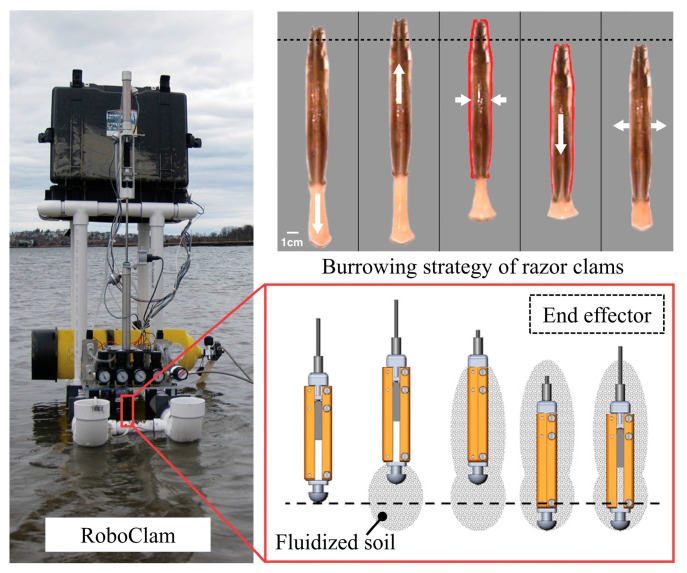
RoboClam and the end effector mechanism [79].

The dual-anchoring movement is a biological movement method similar to wriggling, both involving cycles of radial expansion and axial elongation. The Atlantic razor clam uses this mechanism for its movement in soil. It can penetrate soil with less energy in high-moisture-content soils, using only about 10 N of force to dig to a depth of 70 cm [78]. Winter et al. [79] were inspired to develop a device named RoboClam. Its end effector, in contact with the soil, made up/down and inward/outward movements driven by compressed air, replicating the burrowing kinematics of the Atlantic razor clam. The area around the tip component was fluidized during burrowing, resulting in an exponential reduction in digging energy, as shown in Figure 8. Zhang et al. [80] designed bio-inspired self-burrowing probes. Through discrete element simulation, they validated that these probes effectively mitigated penetration resistance by imitating the dual-anchor locomotion mechanism, thus enhancing penetration efficiency. Soil-engaging components that can achieve wriggling and dual-anchoring movements are usually suitable for soils with a shallow depth and loose texture. They have potential value in engineering applications and can further optimize the design to adapt to a wider range of soil environmental conditions.

The movement mechanisms of animals living in sandy soil are also worthy of study and consideration. Antlions are good at digging holes in the sand. They can swiftly construct conical traps by backing up and vibrating the back of their bodies [81]. Wu and Zhou et al. [82,83] used scanning electron microscopy to obtain the non-smooth surface morphology of antlions and designed a biomimetic subsoiler tip that can reduce draught force. Furthermore, they used a highspeed camera to obtain the vibration digging parameters of the antlion and combined it with the bionic subsoiler shovel. Field results showed that the subsoiler with bionic characteristics, as well as vibration parameters, reduced resistance by 14.2–21.2%. The combination of vibrations and non-smooth surface helped to reduce the soil cohesion and internal friction angle.

**Figure 9:**
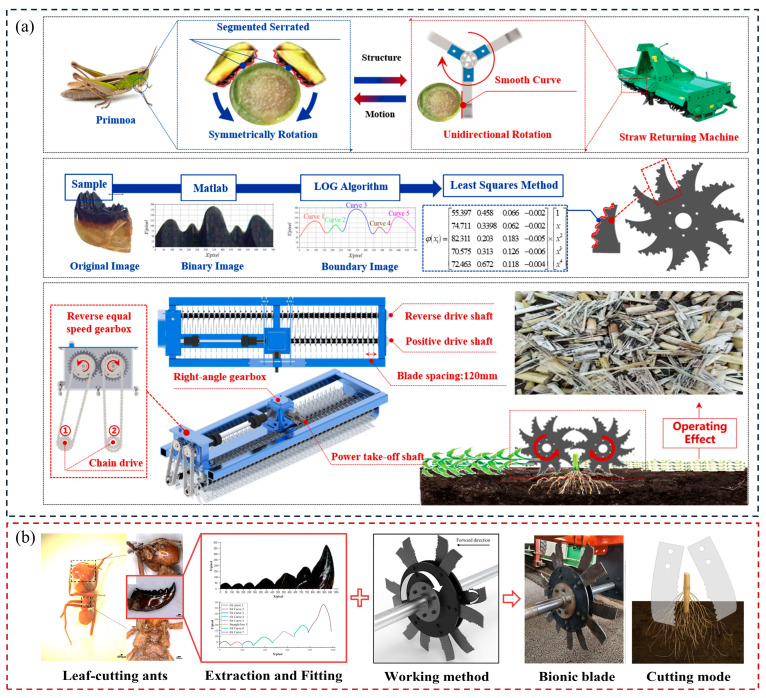
Mouthpart structure and motion inspired soil-engaging components. (**a**) Bionic straw returning blade [87], (**b**) bionic stubble-cutting device [89].

Apart from the movement methods of the animals mentioned above, some other movement mechanisms of animals are equally worthy of our attention and reference, such as the biting movements of insects. The bite mode of the mouthparts of phytophagous insects reflects the characteristics of low resistance and the high efficiency of cutting [84]. Under conservation tillage, there is straw left in the farmland which cannot be effectively cut and destroyed by ordinary cutting tools. Their mouthparts are considered suitable bionic prototypes for straw cutting [85]. Zhao et al. [86] designed a bionic stubble-cutting device based on the biting mode and mouthpart structure of locusts. The device utilizes an isokinetic symmetric cutting method to reduce the horizontal displacement of the stubble. At the same time, it increases the surface stress of stubbles through the bionic multi-segment and serrate structure, which reduces the torque of the imitation cutting by 26.6–31.6% and the power consumption by 21.9–26.1% compared with the common cutting device. The authors have further applied this design approach to the straw returning machine, as shown in Figure 9a. By changing the cutting approach of the stubble return operation from variable to fixed, this method effectively limits stubble displacement and reduces cutting resistance. Consequently, the total cutting energy consumption of the machine is reduced by 9.4–11.7%, while the qualification rate of the crushed length is improved by 10.4–14.7% [87]. Similarly, Zhu et al. [88] designed a disk stubble cutter modeled after the mouthparts of longhorn beetles. This design mimicked the cutting motion of the mouthparts, resulting in lower operational power consumption and a higher cut-off rate.

The root−soil complex in farmland is less likely to be cut than straw. This is attributed to the anchoring effect of roots within the soil, which imparts greater stability and mechanical strength to the root−soil complex. Qi et al. [89] were inspired by the multi-toothed structure and biting mode of the mandibles of leaf-cutting ants and designed the cutter and motion structure of the stubble-cutting device for cutting the root−soil complex, as shown in Figure 9b. Field experiments showed that, at 240 rpm rotary speed, compared to the traditional power straight blade, the torque of cutting was reduced by 15.4%, while the power decreased by 11%. The bionic cutting method of the components plays an important role in the reduction in torque and power consumption.

In addition to animals, plants also exhibit movement behaviors during their growth process. Plant roots demonstrate excellent soil penetration performance as they grow [90]. Mishra et al. [91] designed a probe for soil penetration that imitates plant roots. The root-inspired soft probe incorporates a tip with housing and sloughing elements and multi-chamber soft actuators to simulate the sloughing and radial expansion mechanism of plant roots. The designed bionic probe reduced the penetration resistance by 13.4% and 13.02% energy consumption when penetrating granular soil at 10 mm/min. Chen et al. [92] applied the circumnutations of the root tip to the probe. Discrete element simulation results show that the bio-inspired motion reduced the penetration force and work by changing the soil structure and spreading the force chains around the probe tip. Additionally, the seeds of some flowering plants can bury themselves underground through rotation [93]. Tang et al. [94] studied the penetration resistance of cones at different rotational speeds. Simulation results showed that rotation not only caused a tilt in the contact force but also significantly reduced the magnitude of the contact force and the overall number of contacts. The introduction of rotational motion enables the probe to achieve greater energy efficiency.

#### 2.1.3. Chapter Summary

The relevant characteristics of typical low-resistance biomimetic soil-engaging components are summarized in Table 1 according to the foregoing review, and the following conclusions are drawn:

- The biomimetic design of the morphology and structure is convenient, efficient, and requires minimal alteration to the production process of soil-engaging components, making it the primary method for addressing draught reduction challenges;
- Research on reducing the resistance of soil-engaging components by mimicking organism movement is limited. This is primarily due to the challenges associated with obtaining motion parameters from biological organisms. Factors such as the habits of the organisms and the experimental environment can influence the data accuracy. In addition, moving components require additional energy to drive, provided by fuel or electricity. Such bionic components may result in reduced component resistance but increased total energy consumption, which is worth considering [95]. Nevertheless, the resistance reduction characteristics of biological motion deserve further exploration in this area. They serve as valuable inspiration for the creation of low-resistance components with enhanced performance.
- Scholars have conducted more research on biomimetic draught reduction in soil-engaging components, which represents a current research focus. Mimicking the excellent characteristics of living creatures and applying them to soil-engaging components, it shows a satisfactory performance in tests. The bionic prototypes of bionic low-resistance soil-engaging components are diverse, particularly in regard to soil animals. However, the differences in size and material between these animals and components are enormous. Therefore, the differences in needs and conditions between biological and engineered systems must be carefully considered when designing biomimetic soil-engaging components.

### 2.2. Anti-Adhesion Biomimetic Components

In agricultural and construction machinery operations, the phenomenon of soil adhering to the surface of soil-engaging components is obvious. It increases the energy consumption of machinery and equipment, thereby affecting efficiency. In extreme cases, it can even render the machinery unable to function properly [3]. Creatures in nature also involve adhesion challenges in their survival, especially some soil animals, such as dung beetles [96], earthworms [97], and grubs [98], who spend long periods of time in moist soil but keep their bodies clean. Through long-term evolution, these creatures have gradually acquired excellent anti-adhesion properties. The reasons for resistance to soil adhesion mainly include geometrically non-smooth various morphologies, chemical composition and hydrophobicity, body surface bioelectricity, and the lubrication effect of liquid substances on the surface. Inspired by these soil animals, bionic design has become a new research direction for reducing the soil adhesion of soil-engaging components.

#### 2.2.1. Components Inspired by Biological Non-Smooth Surfaces

Biomimetic studies have shown that most of the organisms in the soil have non-smooth geometrical structures on their body surfaces, which cannot be ignored. These structures often manifest as convex hulls, pits, corrugations, and ribs [99]. They disrupt the distribution of the water film between the organism and the soil surface and decrease the contact area between them, thereby reducing adhesion [100,101].

**Figure 10:**
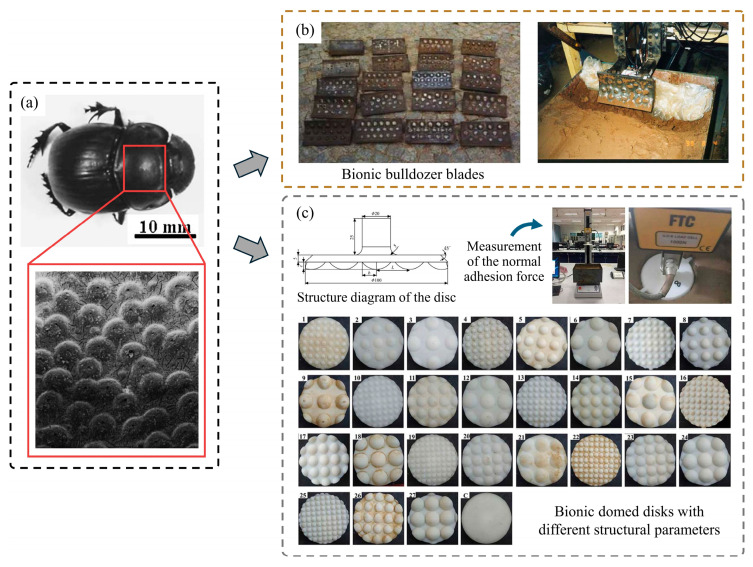
Anti-adhesion biomimetic components with convex structures. (**a**) The convex domes on the pronotum of the female dung beetle [96], (**b**) bionic bulldozer blades [104], (**c**) bionic domed discs [108].

Dung beetles are typical soil animals with a non-smooth structure. Scholars have explored the non-smooth structures found on the pronotum, clypeus, and elytra of dung beetles, which exhibit multiple textures. These distinctive features have been widely utilized as bionic prototypes for designing soil-engaging components suited for different operational situations [96]. Deng et al. [102] fabricated a bionic plow wall with a raised structure on the surface by the hot forging and pressing molding of 65 Mn alloy steel, which showed a significant adhesion reduction effect in clayey soil. Ren et al. [103,104] designed bionic bulldozer blades with non-smooth surface characteristics by imitating the morphology of dung beetles, as shown in Figure 10b. In experiments, the non-smooth blade surfaces exhibited significantly lower soil adhesion, thereby reducing the drag force on the blades. Tong et al. [13,105] developed a bionic press roller based on the geometric structure of the dung beetle’s ventral surface. The bionic pressure roller was found to decrease the contact area with the soil, resulting in a 52.78% reduction in adhered soil compared to the conventional pressure roller. At the same time, due to the improvement of the working effect of the pressing roller, the emergence rate and plant spacing of the plants after the operation have shown beneficial effects. In addition, Gao and He et al. [106,107] were inspired by the convex hull shape of the dung beetle’s head. They, respectively, developed a bionic spiral opener and a bionic spiral bit, which solved the problem of soil adhesion and blockage during the operation of the spiral components.

It is worth noting that the size and arrangement of the convex shapes have a significant impact on reducing soil adhesion. El Salem et al. [108] fabricated twenty-seven discs with convex structures made of ABS based on the Taguchi orthogonal array, as depicted in Figure 10c. The normal adhesion force tests showed that the adhesion force of the domed discs with bionic structures was smaller than that of the flat disc. The adhesion force was mainly related to the height and diameter ratio of the dome. A proper size could significantly reduce the normal adhesion force of the soil.

**Figure 11:**
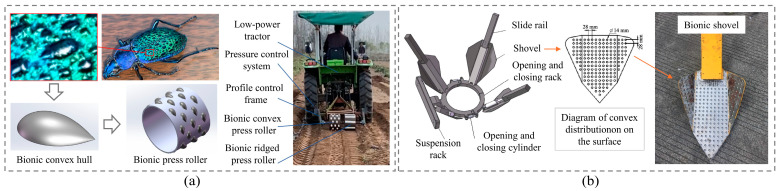
Biomimetic soil-engaging components inspired by Ground beetle. (**a**) Bionic press roller [109], (**b**) bionic tree transplanter shovel [110].

The shells of ground beetles also feature geometrically non-smooth elements. Scholars have used this characteristic to develop bioinspired soil-engaging components aimed at reducing adhesion. For instance, Liu et al. [109] designed a bionic press roller with a convex hull structure, as seen in Figure 11a. In field tests, compared with the ordinary pressure roller, the slip rate of the bionic pressure roller was improved and the soil adhesion was reduced by 51.2%. Similarly, Zhao et al. [110] designed a bionic tree transplanter shovel based on this, as shown in Figure 11b, where the regular arrangement of spheres on the shovel surface reduced soil adhesion.

**Figure 12:**
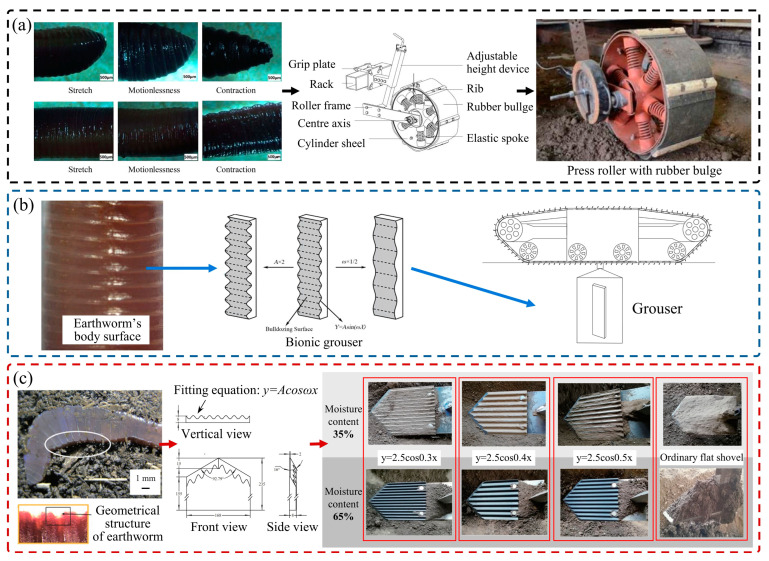
Anti-adhesive soil-engaging components inspired by earthworms. (**a**) Anti-adhesion press roller with rubber bulge structure [111], (**b**) bionic grouser [112], (**c**) bionic potato digging shovel [114].

In addition to the convex hull surface structure, the multi-segmented corrugated shape of the body surface of organisms like earthworms and shells has also been found to have the property of reducing soil adhesion. This corrugated structure can be expressed approximately as a sine or cosine function. Jia et al. [111] designed a bionic press roller, as shown in Figure 12a. By arranging rubber bulge structures of different heights on the roller, the corrugated structure of three surfaces on the earthworm body surface in stretched, motionless, and contracted states was imitated. The test results showed that the soil adhesion of the bionic press roller was reduced by 37.62%. The corrugated structure generated a constantly changing shear force at the soil−component interface during movement, thus reducing adhesion. Ma et al. [112] developed a bionic corrugated grouser based on the structure of earthworms, as shown in Figure 12b, to solve the soil adhesion problem on deep-sea mining machines. The study found that the bionic corrugated grouser with a corrugated structure of *Y* = 2sin(*X*/10) had the best viscosity reduction effect. The bionic structure cut and broke the sticky soil, effectively destroying the internal structure of the soil.

Sticky soil in the field can also hinder the normal operation of the work. Li et al. [113,114] designed a potato bionic digging shovel for sticky and heavy soil. As shown in Figure 12c, the shovel body surface was designed with longitudinal waves. Soil bin and field experiments demonstrated that, when the waveform amplitude (A) was 2.5 and the frequency (ω) was 0.5, the bionic shovels showed a good performance. In the soil condition of 65% high moisture content, as depicted in Figure 12c, the surface of the ordinary flat shovel tended to accumulate more soil, whereas the surface of the bionic digging shovel remained basically free of clay. The existence of the bionic structure made the kinetic energy of soil particles higher, allowing them to pass quickly over the shovel surface, thus reducing soil adhesion by shortening their residence time. Due to the reduction in soil adhesion, the fuel consumption of the bionic digging shovel was reduced by 17.15% under the same conditions. Similarly, the corrugated potato digging shovel designed by Zhao et al. [115] and the wavy coulter developed by Fu et al. [116] also achieved good results in reducing soil adhesion. These studies have provided new ideas and methods to solve the problem of soil adhesion in harvesting machinery. Moreover, for the soft paddy field environment with higher water content, Wang et al. [117] designed a bionic sliding plate for rice direct-seeding machines inspired by the microstructure of the scales on the loach body surface. It effectively addressed the issue of severe adhesion and high sliding resistance experienced by the slide in wet and soft paddy fields.

**Figure 13:**
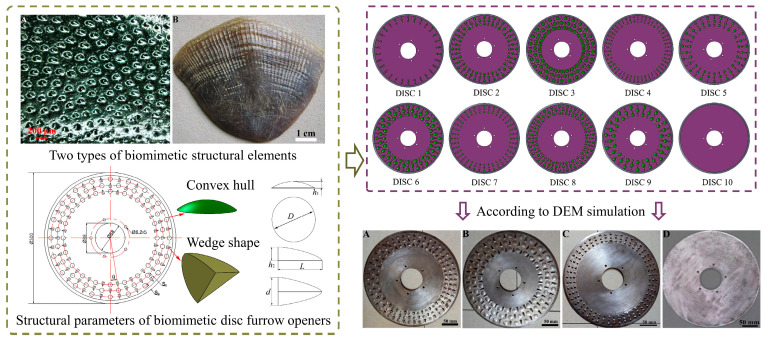
Biomimetic disc furrow opener inspired by two creatures [118].

It is worth mentioning that the source of inspiration for biomimetic soil-engaging components cannot be limited to single bionic prototypes. Wang et al. [118] combined the protruding structure of the pangolin scales and the convex structure of the dung beetle to design nine types of biomimetic disc furrow openers, as shown in Figure 13. They optimized the fabrication of three bionic trenchers with low adhesion and low resistance based on discrete element simulation results. Chen et al. [119] designed bionic penetration heads with pits on the head and evenly distributed grooves around them, which showed good viscosity and drag reduction capabilities. This provided a theoretical reference for designing low-resistance penetration heads and related equipment in trenchless installations. It can be inferred that, by rationally combining multiple bionic elements, the anti-adhesion performance of soil-engaging components can be further improved. Multi-bionic surfaces reduce adhesion resistance by reducing the soil contact area and disrupting the continuous water film between the soils, and the resulting performance improvement is not limited to one aspect. For example, reduced soil adhesion will also reduce the draft resistance of these components.

#### 2.2.2. Components with Coupling Bionic Design

With the deepening of bionic studies, researchers can explore the anti-adhesion properties of organisms from a multidisciplinary perspective. Few biological functions in nature are achieved by a single part or factor. In fact, they are often the result of the synergistic effect of multiple factors [100]. These factors involve morphologies, structures, and materials optimally coupled to form the functional properties of the organism itself. This makes them become the most adaptable systems to the external environment and exhibit maximum coordination [120].

In addition to the special geometric structure of the body surface of soil animals that can reduce soil adhesion, the hydrophobic properties of these surfaces also contribute to their anti-adhesion characteristics. Their body surface has been observed to contain a variety of trace elements, and parts that are in frequent contact with soil contain more Si, S, P, and Al elements [121,122]. Therefore, anti-adhesion can be achieved by imitating the structures and materials of living organisms and using coupling bionic design methods. Polymer materials usually have low soil adhesion and sliding resistance. Their surface hydrophobicity is similar to the surface properties and morphology of animals and plants living in the soil. Among them, ultra-high-molecular-weight polyethylene (UHMWPE) has attracted much attention due to its unique properties, and it has been widely used to solve the adhesion issues between soil-engaging components and soil. By coupling with the bionic structure, the anti-adhesion ability of soil-engaging components is jointly improved. Through coupling with bionic structures, the anti-adhesion capacity of soil-engaging components is synergistically enhanced.

**Figure 14:**
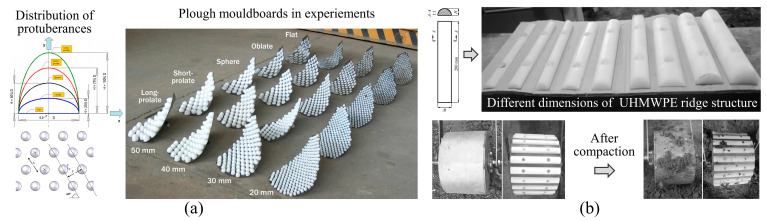
Coupling structural and material of biomimetic soil contact components. (**a**) Bionic plough mouldboards [124], (**b**) ridged bionic press rollers [125].

**Figure 15:**
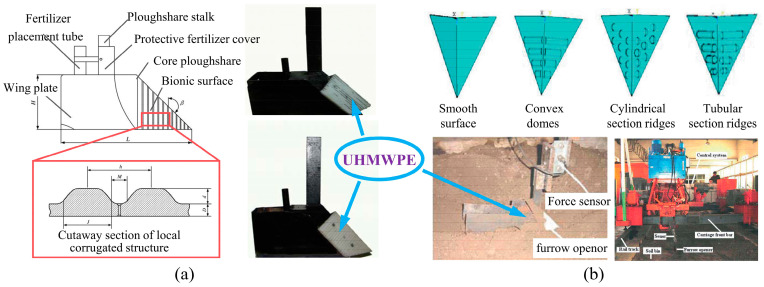
Bionic furrow openers made of UHMPWE. (**a**) Bionic ploughshare [126], (**b**) bionic tine furrow opener [127].

El Salem et al. [123] designed UHMWPE plates with convex structures and tested their anti-adhesion properties at different soil moisture levels and drag speeds. Results showed that, compared to ordinary carbon steel plates, the flat plate with a biomimetic structure showed excellent performance in reducing adhesion and sliding resistance. Soni et al. [124] designed bionic ploughs with different geometries and arrangements by arraying convex domes of UHMWPE on a mouldboard plough, as shown in Figure 14a. Experiments showed that the bionic plough could significantly reduce adhesion and draught force in sticky (37.2% dry basis (d.b.)) and wet (49.1% dry basis (d.b.)) soil. However, not all bionic ploughs displayed an improved performance. When the protuberance height to diameter ratio was exceeded 0.5, the resistance of the bionic plough increased instead. Similarly, Tong et al. [125] designed a biomimetically ridged press roller, as shown in Figure 14b. The bionic structure was achieved by fixing UHMWPE ridge structures onto the surface of the steel press roller. This bionic press roller reduced the contact area with the soil, achieving a maximum adhesion reduction rate of 41.08% in soil bin tests. As illustrated in Figure 14b, only a small amount of soil was attached to the surface of the bionic roller made of UHMWPE after work. However, not all combinations of low surface energy materials with bionic structures effectively reduce adhesion. For example, if the ridge area ratio is too large, it has a negative impact on the performance of the pressing roller. Therefore, the above biomimetic soil-engaging components indicate that reasonable structural dimensions have a significant impact on anti-adhesion performance. In the same way, both Ma et al. and Tong et al. [126,127] used UHMWPE as the material for the non-smooth structure on the surface of soil-engaging components and designed bionic tine furrow openers, as shown in Figure 15. The combination of the low surface free energy of UHMWPE and the corrugated structure to reduce adhesion makes these bionic openers perform satisfactorily during operation. The hydrophobic properties of UHMWPE have a low surface free energy, which reduces the wetting angle of the contact interface between water and soil, thus showing smaller soil adhesion. Although these studies have demonstrated that the synergistic effect of bionic structures and materials has a significant effect on improving the anti-adhesion properties, the considerable disparity in hardness and rigidity between UHMWPE and steel needs to be considered in practical engineering applications.

The combination of bionic structure and surface modification also has obvious coupling and synergistic properties. Jia et al. [128] enhanced the surface hydrophobicity of the bionic bulldozer plate through surface modification. The anti-adhesion effect of the modified bionic bulldozer plate was further enhanced. Zhang et al. [129] developed a biomimetic press roller coated with enamel. Due to the reduction in soil adhesion, its traction resistance decreased by 2.13–22.30% at a speed of 1.04 m/s. Li et al. [130] found that the anti-adhesion performance of the membranous leaf sheath surface was the result of the joint interaction of surface structure and hydrophobicity. Based on this, bionic specimens with various structures were designed. The authors employed myristic acid ethanol solution to modify the surface and decrease its free energy. This bionic structure and modification led to a 50% reduction in the weight of soil adhering to the specimen surface, consequently significantly reducing soil adhesion. It is important to note that there are cost issues in using UHMWPE or coatings in practical applications, and these limitations need to be considered during design.

**Figure 16:**
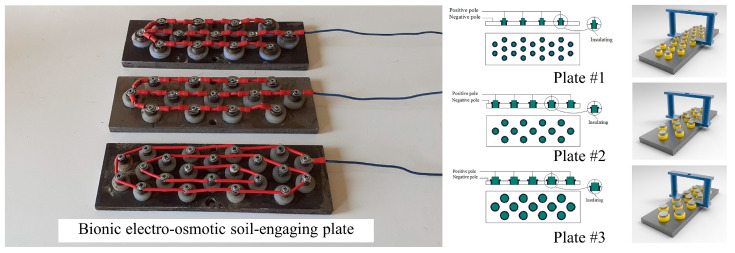
Bionic electro-osmotic soil-engaging component [135].

Some soil animals have been found to have overall electrical penetration on their body surfaces. For instance, during locomotion, earthworms and grubs experience local deformations on their body surfaces due to surrounding soil pressure, leading to the generation of electric potentials [98,131]. This phenomenon demonstrates an ability to resist soil adhesion. Unlike traditional electro-osmotic desorption technology, bionic electro-osmosis places the positive electrode and negative poles on the same surface. This enhances the lubricating contact interface of water, thereby reducing water film tension, viscosity, and adhesive force [100]. Cong et al. [132] conducted experiments on a self-designed electro-osmosis regular test bench and found that increasing the electro-osmosis voltage or time could improve the viscosity reduction effect. Ren et al. [133] applied a bionic electro-osmotic to the surface of a loading shovel and achieved good anti-adhesion effects at a low electro-osmosis voltage of 12 V. Du [134] designed a bionic rotary drilling tool. Under the combined action of the convex hull structure on the drill bucket surface and the optimized electro-osmosis parameters of 24 V and 60 s, the adhesion force was reduced by 65.72%. However, this result was obtained from a drill bucket test conducted under scaled-down and static conditions. Further tests under real-world applications are necessary to determine optimal parameters. Likewise, Massah et al. [135] designed three bionic electro-osmotic soil-engaging plates with different characteristics (Figure 16). When the area of the positive and negative electrodes was 1/4 (plate #3), and a voltage of 24 V was applied for 30 s, the adhesion of the plate was only 0.13 kPa at 31% (d.b.) moisture content. In contrast, the adhesion force of the control non-electro-osmotic plate was 1.41 kPa, which was a substantial reduction in adhesion.

Bionic electro-osmosis can increase the thickness of the water film between the soil−component interface, thereby reducing the adhesion force. However, electro-osmosis requires additional power consumption while reducing adhesion, and the movement state of the components has a greater impact on the viscosity reduction effect [133]. Therefore, the energy saved through electro-osmosis in soil-engaging components should be greater than the additional electrical energy consumed. Otherwise, electro-osmosis is more suitable for components whose structure and materials cannot be easily changed or in environments where the operational effects of components are of greater concern.

#### 2.2.3. Chapter Summary

The relevant characteristics of typical anti-adhesion biomimetic soil-engaging components are summarized in Table 2 according to the foregoing review, and the following conclusions are drawn:

- The non-smooth surface of soil animal bodies has good anti-adhesion properties. Soil-engaging components designed according to the bionic prototype makes the soil−tool interaction on the surface of the components similar to those of the soil animals. Such components effectively reduce soil adhesion by reducing the contact area and disrupting the continuity of the water film;
- Coupling bionics open up new ideas for the design of soil-engaging components that reduce soil adhesion. Coupling bionics combines materials, structures, forms, and functions to achieve synergic effects. Despite the increased complexity, this approach further enhances the anti-adhesion effect, as shown in Table 2. By coupling bionic design methods, the soil-engaging components for a wide range of operating conditions can be developed, which provides a broader perspective for their development.
- Bionic soil-engaging components can reduce soil adhesion, but their effect is often determined by soil conditions and has a limited scope of application. Meanwhile, biomimetic non-smooth structures also place higher requirements on the processing and manufacturing of components. Therefore, it is necessary to conduct further research to improve the applicability and engineering application value of bionic design.

**Table 2:** Summary of anti-adhesion biomimetic components and their related characteristics.

Bionic Prototype	Components	Bionic Design	Components Performance	Ref.
Dung beetle	Bulldozer blade	Convex structure	Adhesion mass↓	[104]
	Press roller	Ridge structure	Adhesion mass 52.78%↓	[105]
			Resistance 28.66%↓	
	Disc	Convex structure	Normal adhesion force 7–18%↓	[108]
Ground beetle	Press Roller	Convex hull structure	Adhesion mass 51.2%↓	[109]
			Slip rate 62.5%↓	
Earthworm	Press roller	Rubber bulge structure	Adhesion mass 37.62%↓	[111]
	Digging shovel	Ripple structure	Soil adhesion↓ Fuel consumption 17.15%↓ Drag resistance 14.45%↓	[114]
Soil animals	Penetration head	Convex/Concave structure	Jacking force 18.7%↓ Adhesion resistance↓	[119]
Dung beetle	Plate	UHMWPE	Adhesion force 10.54%↓	[123]
		Convex structure and UHMWPE	Adhesion force 28.56%↓	
	Mouldboard plough	Flat surface and UHMWPE	Plough resistance 14–22%↓	[124]
		Protuberant structure and UHMWPE	Plough resistance 17–33%↓	
	Furrow opener	Steel ridge structure	Equivalent pressure 13.97%↓	[127]
		Ridge structure and UHMWPE	Equivalent pressure 28.82%↓	
	Drill bucket	Convex structure	Adhesion force 20.18%↓ Adhesion weight 24.04%↓	[134]
		Convex structure and Electro-osmosis	Adhesion force 64.39%↓ Adhesion weight 83.27%↓	
Pangolin scale	Press roller	Polyhedral structure and Enamel coating	Adhesion mass 25.07%↓	[136]
		Polyhedral structure and UHMWPE	Adhesion mass 33.22%↓	
Earthworm	Digging shovel	Ripple structure	Soil adhesion↓ Digging resistance 12.69%↓	[114]
		Ripple structure and Coating	Digging resistance 18.27%↓ Fuel consumption 17.15%↓	
Rhizoma mperatae	Plate	Chemical modification	Adhesion mass 20.02%↓	[130]
		Chemical modification and Bionic texture	Adhesion mass 84.13%↓	

### 2.3. Wear-Resistant Biomimetic Components

Wear is the loss of material caused by the friction of surfaces against each other; it is widespread and undesirable in the production of industries such as agriculture, civil engineering, and mining [137]. In the intricate soil environments where soil-engaging components operate, they encounter hard soil particles, resulting in abrasive wear on their surfaces. This abrasive wear emerges as the primary mode of failure and damage for soil-engaging components, altering their surface shape and edge profile, thereby reducing durability and compromising the machine’s operational quality. Moreover, it leads to additional costs and increases energy consumption [138].

In nature, the body surfaces of many organisms come into direct contact with soil containing mud and sand, inevitably leading to friction during movement. However, the body surface of the organism does not show obvious wear and tear, which was found to be related to the structure of the surface morphology. Many scholars have investigated the abrasive wear-resistant characteristics and mechanisms of these structures, providing new ideas for the design of highly wear-resistant soil-engaging components.

The shells of shellfish typically exhibit a ribbed morphology on their surfaces. Some shells remain in excellent condition after long periods of wear [139]. Ma and Tian et al. [140,141] discovered that the structure of shell surface can change the movement of abrasive particles from sliding to rolling, thereby reducing wear on the surface. Tong et al. [142] used reverse engineering technology to extract the surface structural characteristics of scallop shells and built specimens with bionic ridged surfaces. Abrasive wear tests revealed a 63% reduction in mass loss for the designed bio-inspired ridged surface compared to the flat surface. Notably, the size of the abrasive particles and the groove significantly influenced wear resistance. Similarly, Zhang et al. [143] imitated the surface structure of the shell and designed three types of bionic subsoiler tine samples with ridge structure surfaces. In the test, the wear amount of the bionic tine was less than the flat tine. The wear resistance of soil-engaging components can be greatly increased by applying corrugated structures, including grooves or rib structures, to the structural design of the components.

**Figure 17:**
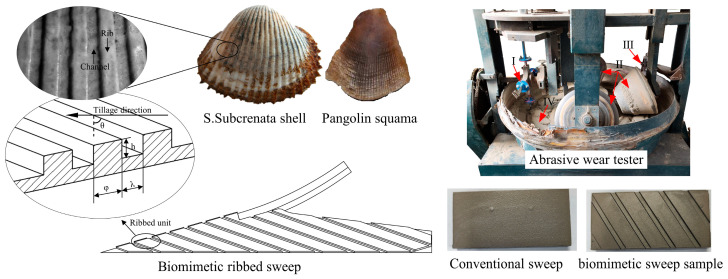
Bionic sweep with lower abrasive wear characteristics [11].

Pangolins have excellent burrowing ability, with scales on the body surface that directly interact with the soil during the digging process. The scale surface is also ribbed and exhibits remarkable abrasion resistance [122]. Zhang et al. [144] designed bionic subsoiler samples based on this structure. In the test, the wear resistance of this sample was 77% higher than that of flat surface samples. The existence of the bionic structure significantly improved the wear resistance. Similarly, Wang et al. [11] designed a biomimetic sweep inspired by the ribbed structures on the surface of the shell and pangolin squama, as shown in Figure 17. Using DEM and response surface methodology (RSM), the dimensional parameters of the biomimetic sweep exhibiting optimal wear-resistant characteristics were determined. Experiments on an abrasive wear tester (Figure 17) found that the bionic sweep showed up to a 34.355% decrease compared to conventional counterparts. Liu et al. [145] prepared samples of bionic non-smooth structures by laser cladding and used disc openers to improve wear resistance. The laser processing technology can efficiently create groove-like non-smooth surface structures, which form a hardened zone surface with dynamic stability and can effectively reduce the wear rate.

**Figure 18:**
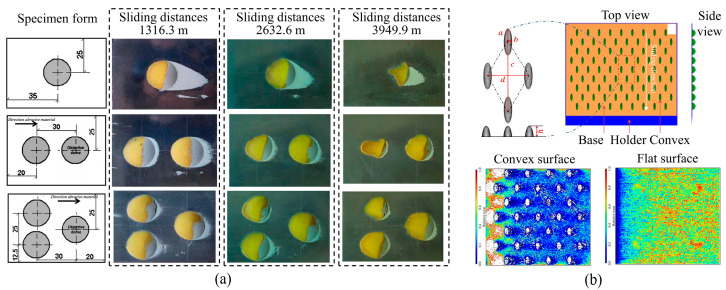
Wear-resistant properties of the concave-convex surface structure. (**a**) Embossed surfaces with convex domes [146], (**b**) DEM simulation study of wear on convex pattern surfaces [147].

Embossed/dimpled surfaces have also been found to be a typical wear-resistant structure on the surface of living organisms. This structure not only reduces adhesion but also exhibits excellent wear resistance. Tong et al. [146] drew inspiration from the convex structures found on the surface of soil-burrowing animals. They fabricated samples featuring arrays of convex domes of various sizes and evaluated the abrasive wear properties of these samples through wear tests using quartz sand particles as the abrasive material. Figure 18a shows the abrasive wear results of the convex dome under different speeds and wear durations. It can be observed that the embossed surfaces guide the movement of the abrasive particles, causing them to wear more on the side of the dome. Similarly, Yan et al. [147] investigated the wear characteristics of surfaces with convex patterns, yielding results comparable to those obtained by Tong et al. The convex surfaces exhibited significantly lower wear compared to flat surfaces, with the majority of wear concentrated at the front half due to the predominant contact of particles with the convex regions in these areas, as shown in Figure 18b.

**Figure 19:**
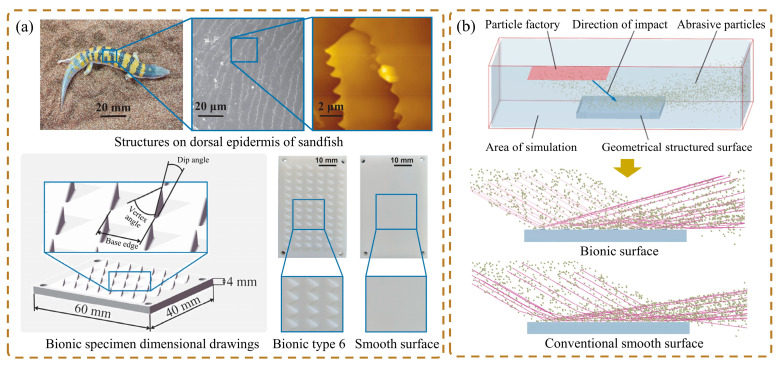
Bioinspired microthorn scale structured surfaces [150]. (**a**) Bionic specimens, (**b**) trajectory of abrasive particles that interacted with surface.

The raised microstructure of some animal body surfaces has also inspired the design of bionic wear-resistant surfaces. Sandfish can move fast under the sand surface in a swimming-like fashion, and the outlines of their heads have low-resistance characteristics [148]. The skin of the sandfish features a low friction coefficient and excellent resistance to abrasive wear, thanks to its surface microstructure effectively countering the wear caused by hard solid particles like sand [149]. Zhang et al. [150] designed and manufactured nine wear-resistant bionic specimens based on the microthorn scale structures of the sandfish epidermis. In the test, it was found that the surface of bionic type 6 with structural parameters of the dip angle of 5°, the basal length of 4 mm, and vertex angle of 120° had the best wear resistance, as shown in Figure 19a. The authors developed biomimetic components based on the microstructure of the cephalothorax exoskeleton of crayfish and also achieved wear-resistant characteristics [151]. In the study of wear behavior, numerical simulations with DEM could effectively simulate particle motion. This method proved to be an effective approach for studying the wear evolution process of soil-engaging components [152]. Through numerical simulation, it was observed that the motion behavior of the particle flow was changed due to the guiding effect of the surface structure. When the particle flow contacted the structure of the microthorn, the rebound angle was larger than the conventional convex hull surface (Figure 19b), which further enhanced the dispersion effect of the particle flow, thereby improving the wear resistance of the bionic surface.

**Figure 20:**
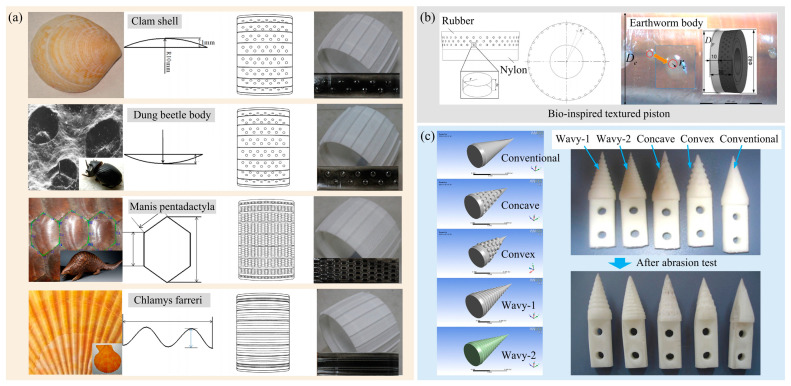
Other biomimetic soil-engaging components with wear-resistant characteristics. (**a**) Bionic wheels [153], (**b**) bio-inspired textured piston for mud pump [156], (**c**) biomimetic anti-abrasion cone form component used for soil cone penetrometer [157].

Based on the above studies, researchers have tried to apply these wear-resistant designs to soil-engaging components across various fields. Non-smooth structures, such as ribs, grooves, convex hulls, and pits, are arranged in an orderly and reasonable manner on the surface of the soil-engaging component. Yan et al. [153] processed the non-smooth structure on the surface of a Mars rover wheel, effectively enhancing its wear resistance and durability, as illustrated in Figure 20a. Compared to the original wheel, the wear mass and energy consumption were reduced by 46% and 26%, respectively. Mao et al. [154] designed a rubber bionic tire featuring a convex-hull structure on the surface. With a convex hull diameter of 3 mm, the wear resistance increased by 9%. Zhang et al. [155] designed a bionic wear-resistant shovel by arranging a groove structure on the outer surface. Likewise, Gao et al. [156] designed bio-inspired pitted pistons with a pit array texture, as shown in Figure 20b. The pit texture can provide lubrication and store abrasive particles, enhancing the wear condition of the piston surface. Additionally, Tong et al. [157] developed a bionic cone component for a soil penetrometer to reduce the wear between the instrument and the soil during testing. As shown in Figure 20c, the surface of the bionic cone was processed with structures such as concave dimples, convex domes, and two wavy forms. The bionic cone with a concave dimpled structure exhibited the best wear resistance.

It is worth mentioning that, besides their application in soil-engaging components, these bionic structures that improve wear resistance have also been extended to many non-soil media in recent years, i.e., bionic drill bits in geotechnical engineering [158,159], bionic turning tools for metal processing [160,161], and bionic brake shoes or discs for vehicle braking [162,163]. All these applications demonstrate significant advantages in wear resistance during operation, underscoring the considerable potential of research on bionic wear resistance.

Based on the above analysis and the data in Table 3, the conclusions are summarized as follows:

- The macroscopic or microscopic structures of organisms have been imitated and applied to the surfaces of soil-engaging components, which has significantly improved their wear resistance. These structures reduce the wear of soil-engaging components by reducing the contact area with abrasives and changing the movement speed and trajectory of abrasives, thereby significantly enhancing durability;
- Wear test is the main method in the current research process of bionic wear-resistant soil-engaging components. However, there is a big gap between the sample materials, abrasives, and wear conditions used in tests and actual working conditions. More comprehensive test methods, together with numerical simulations and field tests, are required to further analyze the performance and mechanism of bionic wear-resistant soil-engaging components;
- When studying bionic wear-resistant soil-engaging components, the impact on other performance aspects requires consideration. The application of bionic structures such as ribs, convex hulls, and pits to soil-engaging components enhances wear resistance but also alters the contact state between the components and the soil. The influence of bionic structures on the structural strength of the components, the resistance and adhesion encountered during operation, and the overall operational effectiveness need further investigation.

## 3. Analysis of Optimizing Performance Mechanisms of Biomimetic Components

When soil-engaging components interact with the soil, the soil will move, flip, and break, and the components will be affected by friction, adhesion, and abrasion. Studies indicate that soil-engaging components inspired by biological properties can effectively enhance their performance. Over the past few decades, extensive research has been conducted by scholars to optimize the performance mechanism of biomimetic soil-engaging components through analytical methods, computer simulations, experimental techniques, and other approaches. Some of these theories elucidate how biomimetic designs operate on components by delving into biological characteristics, while others analyze the efficiency mechanisms of already developed bionic components through practical applications. The main reasons for optimizing component performance are as follows:

### 3.1. Biological Curves and Surfaces

Bionic soil-engaging components are adept at reducing soil cutting resistance effectively. One key attribute contributing to their low resistance is their emulation of the contour curves found in areas where organisms interact with the soil. These curves, characterized by varying curvatures, serve to distribute soil pressure, thereby facilitating easier soil penetration and hole expansion. Research indicates that the two-dimensional outlines of claws and toes across various animals, including mole crickets, moles, badgers, and bears, exhibit relatively similar curvature change patterns. Specifically, these profiles typically feature a larger curvature at the tip and smoother curvature changes towards the middle, aiding in soil penetration and fragmentation [47,164].

Ji and Guo et al. [9,165] investigated the stresses on biomimetic tillage components with variable curvature curves by simulation. Their findings revealed that the leading section of structures with variable curvature characteristics induces continuous stress fluctuations ahead of the component surface. This effect not only effectively reduces the friction between the soil and the component but also loosens and breaks the compacted soil. Similar soil fluctuation phenomena were observed with the bionic bulldozing plate [166]. In contrast, the curves on mouthparts of insects such as locusts and ants differ from those of soil animals, being mostly serrated. The curvature of these tooth-like structures varies over a broader range, resulting in soil-engaging components bearing greater pressure per unit area. This maximizes soil stress concentration, enhances the soil crushing effect, and improves the cutting and fracturing of soil [89].

Soil-engaging components also achieve low-resistance properties by imitating the curved surfaces of biological organs. These bionic surfaces influence the soil movement by changing the direction of the force on the soil. Simultaneously, stress along the excavation direction is dispersed, enhancing soil fluidity on the component’s surface. For instance, the inner curved surface of mole rats’ and mole crickets’ claw toes, serving as the primary working surface, facilitates the smooth movement of excavated soil, preventing accumulation, and reducing cutting resistance [9,167]. Similarly, in using a bionic digging shovel with the characteristics of a mole claw toe, it was observed that the bionic macro-curved surface could effectively guide soil movement and change the stress state of the soil, making it easier to break [32]. Furthermore, simulations of a bionic subsoiler tine, emulating the curved surface of a sandfish’s head, showed improved soil fluidity and reduced soil accumulation, thus reducing the draft force of the tine [148].

Overall, it is effective to apply bionic curves or surfaces to optimize the performance of soil-engaging components. The contour features of variable curvature make it easier for parts to enter the soil, and the guidance of bionic curved surfaces improves soil particle flowability. These bionic designs, inspired by biological structures, effectively reduce the resistance of soil-engaging components and enhance their operational performance.

### 3.2. Non-Smooth Surface Structures

Biomimetic soil-engaging components typically enhance performance by arranging texture structures such as corrugations, pits, and convex hulls on their surfaces. These geometric features change the interaction state between the soil and the contact interface, affecting the movement of soil particles, thereby reducing adhesion between the component and the soil or improving the component’s wear resistance.

#### 3.2.1. Contact Condition between Biomimetic Component and Soil

Biomimetic non-smooth surface structures can reduce the contact area with soil. When non-smooth surface structures are arranged reasonably, soil with a certain stiffness will be supported by the top of the structure on the surface of the component. Compared to smooth surface components, a biomimetic non-smooth surface can reduce the contact area, thereby decreasing the occurrence of adhesion. This has been confirmed by the research of El Salem et al. [108]. They conducted normal adhesion force tests on discs with different convex structures and found that appropriate convex sizes and arrangements can significantly reduce the normal soil adhesion force. Soil with low moisture content appears granular. When the size of soil particles is larger than the spacing between non-smooth structures, the particles cannot directly contact the bottom, resulting in reduced contact area and decreased adhesion occurrence. Certainly, under static conditions, non-smooth surfaces may increase the actual contact area with the soil. However, in practical operation, soil-engaging components typically undergo relative motion with the soil. Therefore, when the non-smooth structure is combined with suitable soil conditions, it can effectively reduce adhesion.

**Figure 21:**
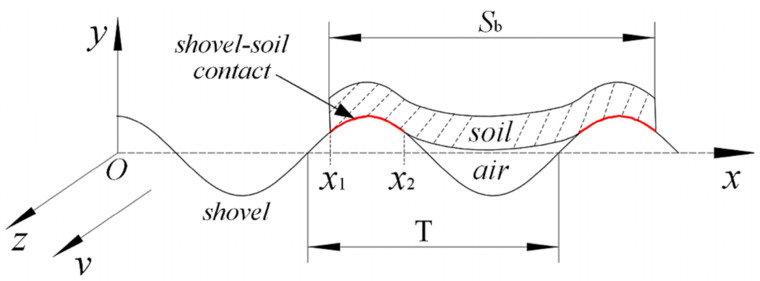
Theoretical diagram of contact between bionic structures and soil [113].

The adhesion of the components touched is closely related to the morphology of the water film between the contact interfaces [168]. When the soil moves on a non-smooth structure, the action of the raised part can disrupt the continuity of the water film and thus reduce the soil adhesion, as shown in Figure 21. In addition, the non-smooth structure can also form small gaps between the components and the soil. These gaps can store air and effectively diminish the negative pressure effect of the atmosphere on the soil. According to the mathematical derivation by Jia and Li et al. [99,113], it is theoretically established that a higher ratio of amplitude to the period of the corrugated non-smooth surface enhances surface hydrophobicity. This makes it easier to form a composite interface between the soil and the non-smooth surface, resulting in a more pronounced reduction in soil adhesion. Consequently, some soil-engaging components fabricated from low surface energy materials with non-smooth structures perform better in terms of anti-adhesion and draught reduction.

#### 3.2.2. Soil Particle Movement on Biomimetic Component Surface

**Figure 22:**
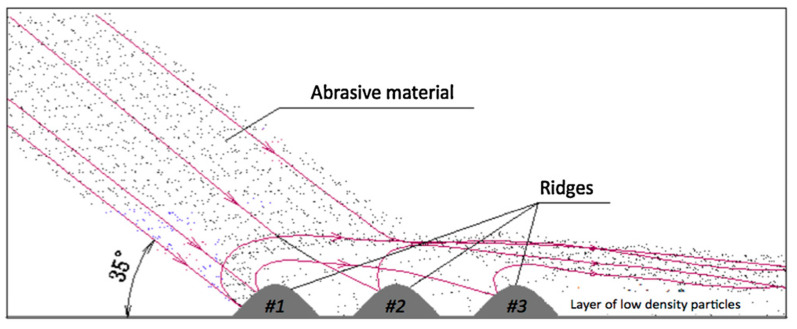
Soil particle motion field on the surface of biomimetic structures [146].

One reason why bionic structures reduce surface abrasive wear is their alteration of particle velocity and trajectory on the component’s surface, thereby decreasing the velocity of soil particles. The ribbed and convex hull structure allows more particles to move from sliding to rolling, reducing the micro-ploughing caused by hard particle movement on the contact surface, thereby reducing wear on the component surface. Tong et al. [142,146] proved the effectiveness of rolling and guiding effects through a combination of discrete element simulations and experiments. Abrasive particles bounce off the structure of the biomimetic non-smooth surface, deflecting from the flow direction of the abrasive material. In the process of moving away from the surface, it will also collide with the mainstream particles, making the movement trajectory smooth and reducing the abrasive speed and energy, as depicted in Figure 22. Both convex-hulled and ribbed surfaces yield similar effects, but the convex hulls will also provide a symmetrical guidance effect that is effective for abrasive flow in any direction [146].

**Figure 23:**
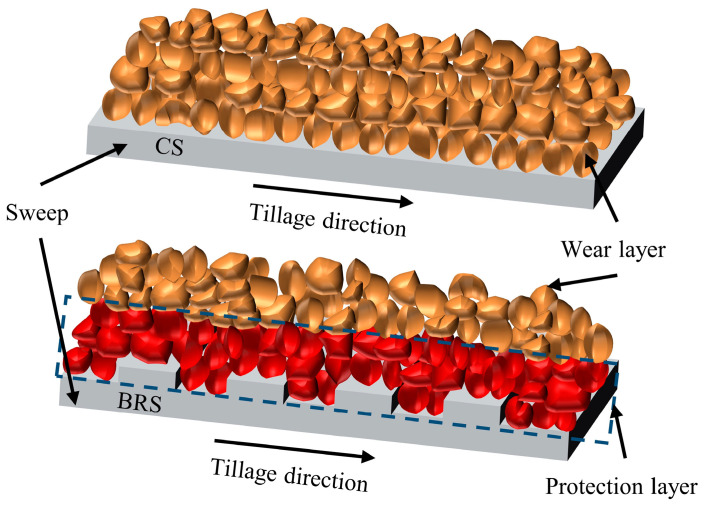
Mechanism of soil particles delamination leading to wear reduction [11].

Another factor contributing to improved wear resistance is that the biomimetic structure can induce interference among abrasive particles. Some particles separate when moving on a non-smooth surface, forming a protective layer, as shown in Figure 23. This alteration shifts the wear process from between soil and components to between soil particles, reducing the contact area and stress between the abrasive particles and the component surface. This ultimately leads to a decrease in wear on the component surface. In addition, whether it is a corrugated structure, a convex hull structure, or a concave structure, a semi-enclosed space can form between adjacent features. Hard particles in the soil can be stored or form a vortex layer of abrasive material. These effects decrease the probability and strength of contact between the abrasive material and the surface, resulting in better wear resistance compared to conventional flat surfaces [169].

For the bionic non-smooth surface formed by laser processing, phase transformation hardening of the metal structure usually occurs, creating an alternating soft and hard bionic non-smooth structure. This high-hardness zone prevents the abrasive particles from penetrating the matrix material, cutting off the plough and peeling effect of the abrasive particles on the material and hindering the wear process during the wear process, thereby improving the wear resistance. Additionally, the non-smooth surface provides better guidance for particles and reduces wear.

In summary, the adoption of biomimetic design effectively improves the draught reduction, adhesion reduction, and wear resistance of soil-engaging components, thereby improving operating results. Certainly, the bionic design methods are not only limited to these three areas alone and can enhance the performance of soil-engaging components in other aspects. For instance, biomimetic tires and tracks exhibit high trafficability in dry sand or sticky−wet ground conditions [170,171], while biomimetic walking feet demonstrate a high load-bearing capacity on soft terrain [172,173]. Although there is currently no unified discussion on the optimal performance mechanism of bionic soil-engaging components, we can conduct in-depth and comprehensive research from a multidisciplinary perspective. Exploring organism structures, distribution patterns, environments, and movement patterns can further advance the development of bionic soil-engaging component design.

## 4. Conclusions and Outlook

In recent years, significant progress has been made in the biomimetic design of soil-engaging components. These biologically based studies are inspiring the optimization and improvement of soil-engaging components. Examples include low-resistance components designed by imitating the clawed toes of digging animals, as well as anti-adhesion and wear-resistant components that draw on features of biological non-smooth body surfaces. This paper provides a comprehensive summary of bionic prototypes for soil-engaging component design along with a review of current research and application progress. Finally, it concludes by examining the mechanism through which biomimetic soil-engaging components enhance performance, presenting the following main conclusions and outlook:

(1) The biomimetic design of soil-engaging components is effective, and a large number of organisms provide inspiration for the bionics. However, current research on bionic principle is still insufficient, with most designs relying on methods such as the similarity principle, rule of thumb, and experiments. For instance, the scale of bionic design presents a challenge, as biological surface structures span from nanometers to millimeters, posing a significant gap compared to conventional soil-engaging components in engineering. Reasonable methodologies are needed to support these studies. In future research, the functional mechanisms of bionic prototypes can be studied through more advanced testing techniques and professional characterizations. By investigating the key mechanisms underlying draught reduction, anti-adhesion, and wear resistance, a more solid theoretical foundation can be established for the design of biomimetic soil-engaging components.
(2) Bionic coupling design is a future development trend. It is a method that is more comprehensive and closer to the function of biological prototypes and can further improve the performance of soil-engaging components. Existing bionic designs tend to imitate a single feature. However, there is a huge gap between single research and biological entities, and bionics in a single dimension alone cannot lead to perfect results. The requirements for soil-engaging components have become more complex and stringent with human exploration and economic development. Coupling bionics leads to a shift in the design of soil-engaging components from simply imitating biological structures to a deeper level of understanding, realizing a higher level of performance improvement and engineering applications. Through coupling bionics, more effective solutions can be achieved for intricate engineering challenges.
(3) Computer simulations are essential for selecting, designing, and optimizing biomimetic soil-engaging components. Traditional testing is often constrained by factors like equipment availability, soil conditions, and climate. Simulation offers flexibility to overcome these limitations, providing significant convenience for optimizing biomimetic soil-engaging components. In subsequent research, efforts can be directed towards establishing more accurate simulation models. Examples include improving the accuracy of component models and using soil models that better match actual conditions. Additionally, various calculation methods, including the discrete element method (DEM), finite element method (FEM), multibody dynamics (MBD), and computational fluid dynamics (CFD), offer unique advantages. Combining these methods enables more accurate simulation of realistic scenarios for analyzing biomimetic soil-engaging components. For example, combining DEM and MBD allows for the study of the operating effects of complex moving components [71], while combining CFD and DEM allows for the exploration of soil−tool interaction mechanisms in high-moisture-content soils [174]. These methods help to observe more details of soil movements and interactions, enhancing the reference value of simulation results.
(4) Biomimetic soil-engaging components have been proven to have excellent performance, but their widespread adoption in engineering remains limited. The intricate contours and structures of these components often require precise machining methods and additional processing steps, leading to increased production and maintenance costs. In addition, biomimetic soil-engaging components may improve performance in one aspect but have a negative impact on another. For example, adding groove structures may weaken the component’s strength. Therefore, it is necessary to use advanced manufacturing technologies and materials to find a balance between the component design, durability, and manufacturing costs, facilitating broader adoption of biomimetic soil-engaging components in practical operations.

This article provides a review of the biomimetic design of soil-engaging components. With the enrichment of new bionic prototypes, the gradual clarification of bionic mechanisms, and the continuous innovation of bionic methods, it is believed that the biomimetic application of soil-engaging components will achieve better results. The development of high-performance soil-engaging components characterized by reduced energy consumption and enhanced operational efficiency is expected to bring numerous opportunities for innovation and advancement in fields such as modern agriculture and civil engineering.

## Figures and Tables

**Table 1:** Summary of low-resistance biomimetic components and their related characteristics.

Bionic Prototype	Components	Methods	Working Parameter	Components Performance	Ref.
Mole cricket (forefoot)	Subsoiler	DEM simulation	Crank speed 100 rpm	Horizontal resistance 16.34%↓ Vertical resistance 24.53%↓ Energy consumption 9.64%↓	[8]
	Rotary blade	DEM simulation	Rotary speed 300 rpm Forward speed 0.66 m/s	Power consumption 14.62%↓	[24]
Mole rat (claw)	Rotary blade	Field test	Rotary speed 220 rpm Forward speed 0.5 m/s Tillage depth 120 mm	Torque 3.91%↓ Tillage depth stability > 93.59%	[28]
	Rotary blade	Field test	Rotary speed 300 rpm Forward speed 0.1 m/s Tillage depth 70 mm	Torque 21.05%↓ Power requirement↓	[29]
	Cutting disc	FEM simulation	Rotary speed 300 rpm Forward speed 1.5 m/s	Combined stress 34.33%↓ Normal stress 22.64%↓	[26]
	Disc	Soil bin test	Forward speed 1 km/h	Mean vertical resistance 21.4%↓ Mean draught force 28.7%↓	[30]
	Disc	Field test	Speed 0.1 m/s Tillage depth 40 mm	Draught force 22.8%↓	[31]
	Digging Shovel	Field test	Forward speed 0.56 m/s Digging depth 250 mm	Draught force 13.33%↓ Fuel consumption 9.18%↓	[32]
	Rotary blade	Soil bin test	Rotary speed 160–320 rpm Forward speed 3 km/h Operating depth 80 mm	Mean torque requirement 13.99%↓	[34]
	Subsoiler	Field test	Forward speed 1.8 m/s Tillage depth 460 mm	Draft force 18.61%↓ Soil looseness 20.6%↑	[35]
Mouse (claw)	Subsoiler	Soil bin test	Forward speed 0.192 m/s Tillage depth 350 mm	Horizontal resistance 7.1%↓ Vertical resistance 24.0%↓	[36]
	Subsoiler	Field test	Forward speed 2 km/h Tillage depth 350 mm	Draft force 8.9%↓	[38]
Badger (claw)	Cutter teeth	FEM simulation	Rotary speed 450 rpm Forward speed 1.5 km/h Cutting depth 50 mm	Cutting resistance↓ Kinetic energy↑	[10]
	Bucket teeth	Wedging test	Cutting speed 1 m/min Cutting depth 40 mm	Buried resistance 12.6%↓	[41]
Badger (teeth)	Specimen	FEM simulation	Cutting speed 4 mm/s Penetration depth 50 mm	Penetration force 26.15%↓	[47]
	Furrow opener	Soil bin test	Operating speed 7.2 km/h Operating depth 50 mm	Working resistance 8.71%↓	[48]
Bear (claw)	Plowing device	DEM simulation	Rotating speed 260 rpm Forward speed 0.3 m/s Plowing depth 128 mm	Power consumption 34.9%↓ Specific energy consumption 5.22%↓	[49]
Anteater (claw)	Shovel	Field test	Forward speed 0.83 m/s Tillage depth 250 mm	Tillage resistance 8.11%↓ Soil disturbance 20.26%↓	[52]
Shark (skin)	Subsoiler	Soil bin test	Cultivation speed 2.1 km/h. Depth 200 mm	Horizontal resistance 21.3%↓ Vertical resistance 24.8%↓	[55]
	Subsoiler	Soil bin test	Operation speed 3.6 km/h Tillage depth 400 mm	Tillage resistance 60.4%↓	[57]
Mole (digging)	Subsoiling mechanism	DEM simulation	Rotational speed 100 rpm Forward speed 0.5 m/s	Drag resistance 18.4%↓ Soil disturbance 312.78%↑	[65]
	Cleaner	DEM simulation	Forward speed 2.22 m/s	Working resistances 49.4%↓ Straw cleaning rate 11.2%↑	[66]
Hare (digging)	Profiling energy storage device	Field test	Operating velocity 4–5 km/h Tillage depth 400 mm	Fuel consumption 16.1%↓ Soil disturbance↓	[71]
Earthworm (wriggling)	Probe	Penetration test	Penetration speed 2 mm/s Penetration depth 100 mm	Penetration resistance 80%↓	[76]
Antlion larva (vibration)e	Subsoiler	Field test	Forward speed 3–7 km/h Tillage depth 300 mm	Working resistance 14.2–21.2%↓ Energy consumption 11.2–16.5%↓	[83]
Locust (biting)	Cutting blade	Soil bin test	Rotation speed 930 rpm Forward speed 6 km/h	Cutting energy 9.4–11.7%↓ Power consumption 10.4–14.7%↓	[87]
Ant (biting)	Cutting blade	Field test	Rotation speed 240 rpm Forward speed 3 km/h	Cutting torque 15.4%↓ Power consumption 11%↓	[89]
Root (growing)	Probe	Penetration test	Penetration speed 10 mm/min Penetration depth 150 mm	Penetration resistance 13.4%↓ Energy consumption 13.02%↓	[91]

Notes: ↓ indicates a decrease; ↑ indicates an increase. These symbols are used consistently in the following tables.

**Table 3:** Summary of wear-resistance biomimetic components and their related characteristics.

Bionic Prototype	Components	Working Parameter	Components Performance	Ref.
Shell	Ridged surface	Sliding distance 82 m	Mass loss 63%↓	[142]
	Subsoiler tine	Sliding speed 3.02 m/s Sliding distance 4100 m	Mass loss 30%↓	[143]
Shell/Pangolin quama	Subsoiler sample	Sliding speed 1.68 m/s	Mass loss↓ Wear resistance 77%↑	[144]
	Sweep	Wear speed 2 m/s Wear distance 6427.2 m	Mass loss 34.355%↓	[11]
Sandfish	Specimen	Wear distance 11,400 m	Mass loss 72.7%↓	[150]
Earthworm	Mud pump piston	Rotating speed 2.67 rpm Pressure 0.1 MPa	Mass loss↓ Temperature value↓	[156]
Non-smooth morphology	Wheel	Wear time 40 h	Mass loss 46%↓ Energy consumption 26%↓	[153]
	Tire	Applied force 750 g Friction distance 40 m	Wear resistance 9%↓ Sliding ability 3.4% (sand)/4.2% (glass)↑	[154]
	Cone	Wear speed 2.35 m/s	Mass loss 54.04%↓	[157]

## Data Availability

Data sharing is not applicable to this article as no new data were created or analyzed in this study.
